# The Clinical Uses of Electrocochleography

**DOI:** 10.3389/fnins.2017.00274

**Published:** 2017-05-19

**Authors:** William P. Gibson

**Affiliations:** The Sydney Cochlear Implant Centre, University of SydneyGladesville, NSW, Australia

**Keywords:** transtympanic EcochG, auditory threshold, endolymphatic hydrops, auditory neuropathy, intraoperative EcochG, Perilymph fistula, intracochlear EcochG

## Abstract

The clinical uses of electrocochleography are reviewed with some technical notes on the apparatus needed to get clear recordings under different conditions. Electrocochleography can be used to estimate auditory thresholds in difficult to test children and a golf club electrode is described. The same electrode can be used to obtain electrical auditory brainstem responses (EABR). Diagnostic testing in the clinic can be performed with a transtympanic needle electrode, and a suitable disposable monopolar electrode is described. The use of tone bursts rather than click stimuli gives a better means of diagnosis of the presence of endolymphatic hydrops. Electrocochleography can be used to monitor the cochlear function during surgery and a long coaxial cable, which can be sterilized, is needed to avoid electrical artifacts. Recently electrocochleography has been used to monitor cochlear implant insertion and to record residual hearing using an electrode on the cochlear implant array as the non-inverting (active) electrode.

The electrocochleogram (EcochG) reveals the electrical potentials derived from the cochlea. It is the equivalent for the ear of the electrocardiogram for the heart but it has been largely neglected by clinicians as it can be difficult to obtain unless minor invasive surgery is undertaken. Non-medical clinicians cannot legally undertake the surgery and medical clinicians may not choose to expend their time on a minor procedure.

## The basic electrocochleography potentials

There are three basic potentials: the action potential (AP), the cochlear microphonic (CM) and the summating potential (SP).

The action potential (AP) is derived from the afferent cochlear nerve fibers as they enter the habenula perforate. The EcochG records from a cluster of nerve fibers depending on the frequency of the stimulus. The click stimulus will activate the entire length of the cochlea but it is governed by the speed of the traveling wave which starts rapidly at the basal end (approximately 30 m/s) and then slows down along the cochlear partition as it reaches the apex of the cochlea (approximately 1 m/s). As the click AP is the algebraic summation of the individual AP and the compound waveform is mostly composed of the nerve fibers that fire closely together. The click AP is usually centered on a frequency of 3.2 k Hz. The tone pip AP [compound action potentials (CAP)] are derived from different portions of the cochlear duct and provide some level of frequency specific information.

The cochlear microphonic (CM) is derived from the movement of the hair cells. The waveform resembles the electrical form of the stimulus. If the recordings are derived from outside the cochlea, the CM can easily be confused with an artefactual microphonic. There is no true threshold for the CM as this depends on the quality of the recording apparatus. CM recorded from inside the cochlea, through a cochlear implant, is much less likely to contain artifact. Clinically it may be utilized to show immediate changes during cochlear implant electrode insertion.

SP is a DC potential arising in response to an AC stimulus. Thus the potential is a non-linear response which results when a generator produces more electrical potential in one polarity rather than the other. There are many potential sources but the dominant source is the non-linear vibration of the basilar membrane at higher stimulus intensities and this causes an unequal output of the CM.

## Hearing testing

Electrocochleography was initially utilized as an objective hearing test for young children (Ruben et al., 1960) but has mostly been replaced by less invasive tests. Hearing is a subjective phenomenon and EcochG only tests the threshold of the cochlear CAP. Nevertheless the cochlea as the usual site of dysfunction, it usually related directly to the auditory threshold.

Threshold CAP can only be obtained using a transtympanic electrode and young children require a general anesthetic. Babies can be tested during natural sleep or sedation using auditory brainstem responses (ABR), steady state evoked potentials (SSEP), and cortical auditory evoked potentials (CAEP). EcochG testing is now only used for older children who usually have other disabilities such as autism and marked developmental delay making behavioral testing unreliable. EcochG testing can also be undertaken during another procedure such as insertion of ventilation tubes. One advantage of EcochG is that no masking of the opposite ear is needed.

### Technical aspects

The usual method involves inserting a transtympanic needle electrode through the eardrum to lie close to the round window. If there is a cochlear malformation, there is a danger of penetrating an abnormal round window and causing a gush of perilymph. The needle electrode has a high input impedance (40–50 kΩ) and this can act as a high pass filter excluding the some of the frequencies needed to collect CAP at 500 Hz and lower.

**Figure 1:**
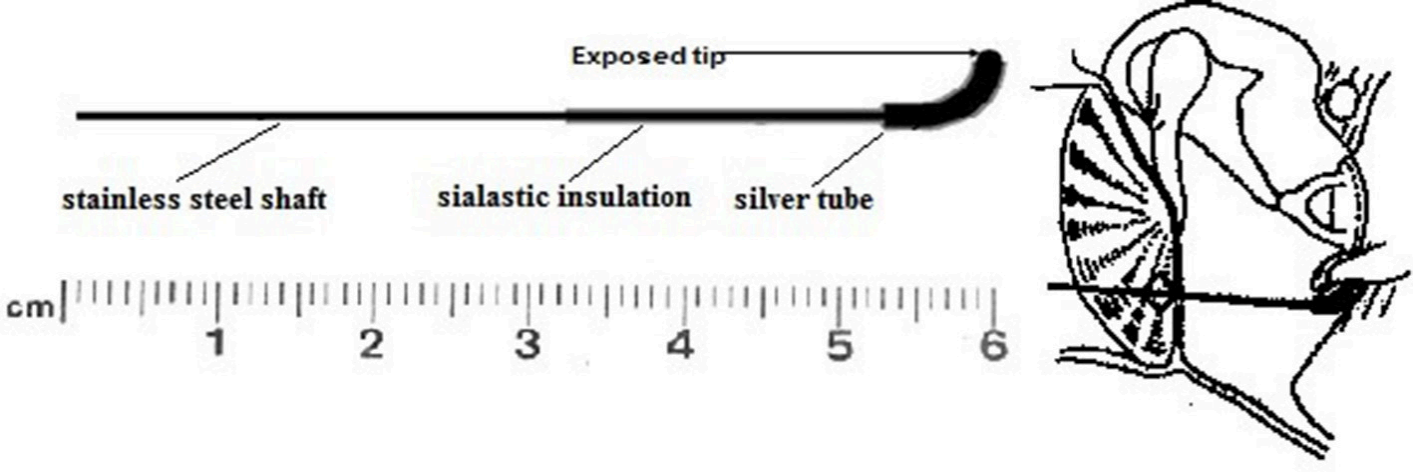
**The “golf club” electrode**.

The author has developed a “golf club” electrode with a blunt end which is inserted by the surgeon through a posteriorly placed myringotomy incision with a view of the round window niche (Aso and Gibson, 1994; Figure 1). This electrode avoids inadvertent damage to the round window, yields larger CAP especially at the lower audiometric frequencies, and can be utilized to obtain electrically evoked ABR (EABR) (Walton et al., 2008). The usual protocol is to test using stimuli at 500 Hz, 1 kHz, 2 kHz, 4 kHz, and 8 kHz. It is possible to test at 250 Hz but due to the lack of synchrony, the response is very wide and it is difficult to get clear thresholds.

### Auditory neuropathy spectrum disorder (ANSD)

**Figure 2:**
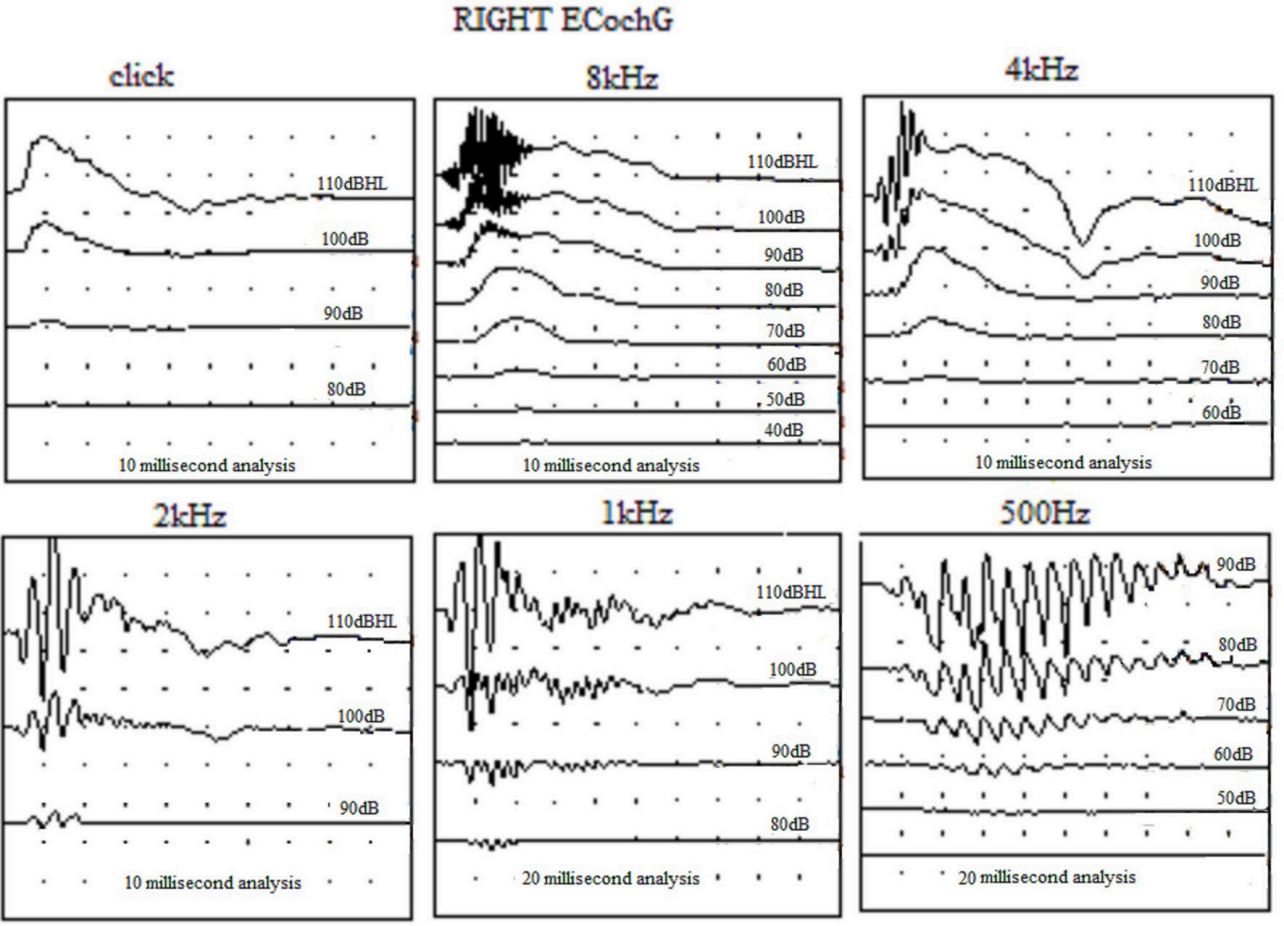
**Electrocochleography (ECochG) traces from an ear affected by auditory neuropathy**.

**Figure 3:**
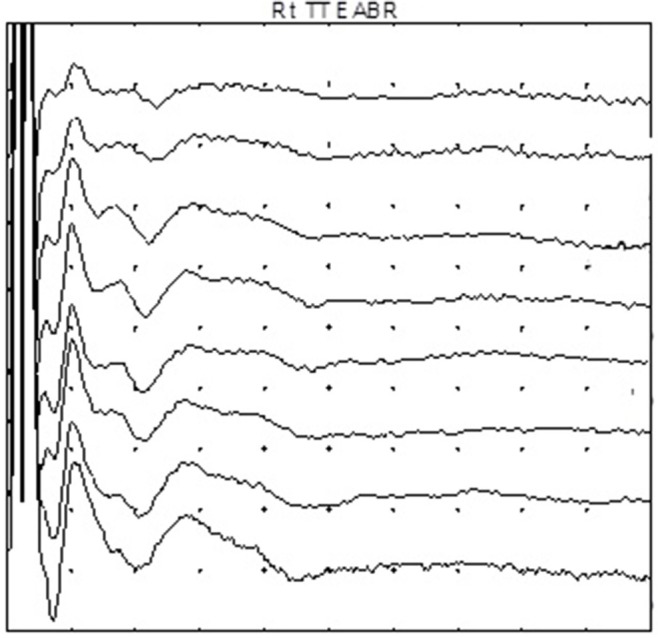
**The electric auditory brainstem responses (EABR) obtained from an ear with auditory neuropathy due to perinatal asphyxia**.

The term “auditory neuropathy” may suggest an underlying neural pathology but EcochG and EABR suggest an underlying cochlear pathology which can exist with or without any neural dysfunction. EcochG shows a greatly enhanced CM which causes an abnormal positive potential (APP) at 8, 4, and 2 kHz and at lower audiometric frequencies there is a broad negative distorted waveform (Figure 2). ABR may only show an initial positive deflection which has been mistaken for N1 with the lack of the ensuing waveform. EABR usually shows a completely normal EABR waveform (Figure 3) and these ears have excellent results after cochlear implantation. In a few cases, especially when the MRI fails to reveal a separate cochlear nerve, the EABR are absent or distorted and cochlear implantation usually yields a poor outcome (Walton et al., 2008).

The APP shows no relationship with the audiometric threshold and can lead to errors using SSEP. The likely explanation is that there are surviving outer hair cells generating CM despite the loss of inner hair cells (Gibson and Graham, 2008). The outer hair cells distort the tuning of the basilar membrane affecting the output of the remaining inner hair cells leading to poor speech discrimination using conventional hearing aids. As the cochlear nerve is usually unaffected these ears perform well with cochlear implants and may be considered when audiometry only suggests a moderate hearing loss.

## Diagnostic testing

Electrocochleography can help the clinician to differentiate different pathological conditions. The most useful is the diagnosis of endolymphatic hydrops which is present in Meniere's disease and other less common conditions.

### Technical aspects

**Figure 4:**
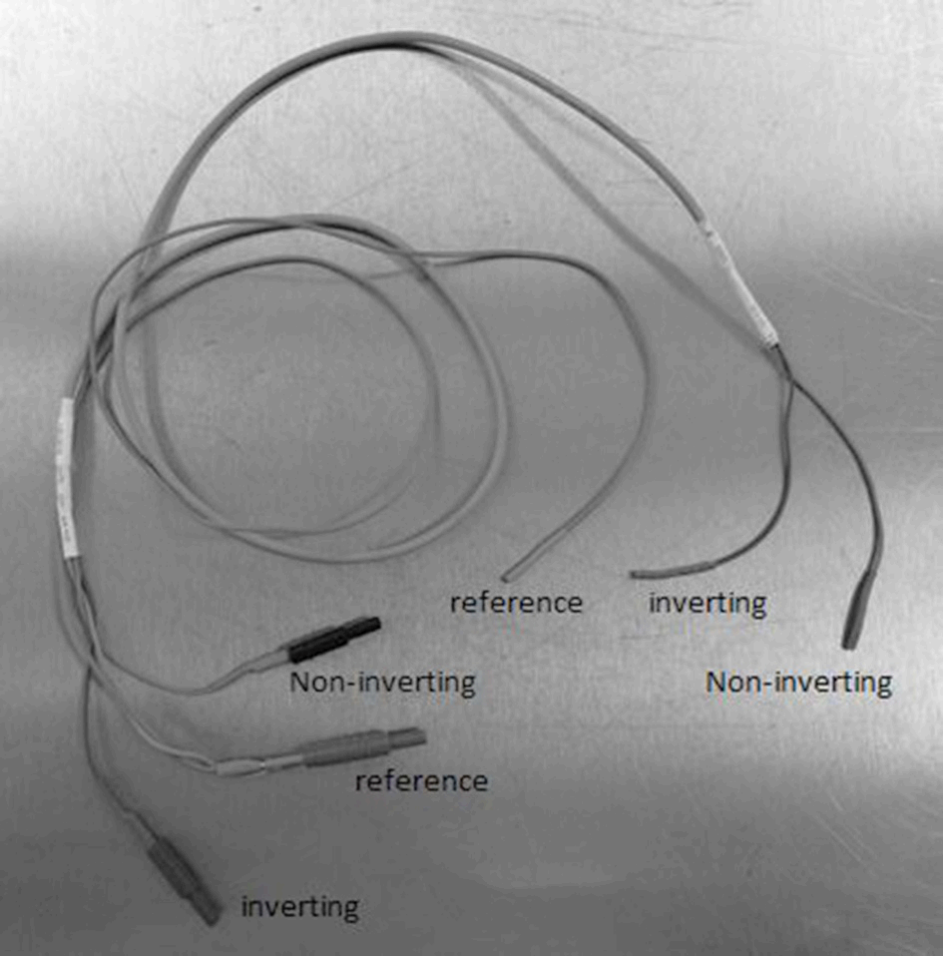
**The long coaxial cable which connects the electrodes to the preamplifier which can be sterilized for intraoperative recordings**.

Using a transtympanic needle provides larger and more robust recordings than the extratympanmic electrode placement. The surgeon can anaesthetise the tympanic membrane using a droplet of phenol or and an anaesthetic cream. The author has not had any personal mishaps placing the needle in adults although care must obviously be taken not to contact the stapes and no persistent perforations have occurred. The amplifier settings must allow some DC input and the author uses a bandpass of 3.2 Hz to 3 kHz. Electrical interference can be problematic in hospital settings and the author uses very short lead on the inverting and non-inverting electrodes plugged in to a co-axial cable which reaches the preamplifier (Figure 4). The author uses a Teac® disposable monopolar electrode and removes the plastic end so it can fit into the needle holder.

#### Meniere's disease

**Figure 5:**
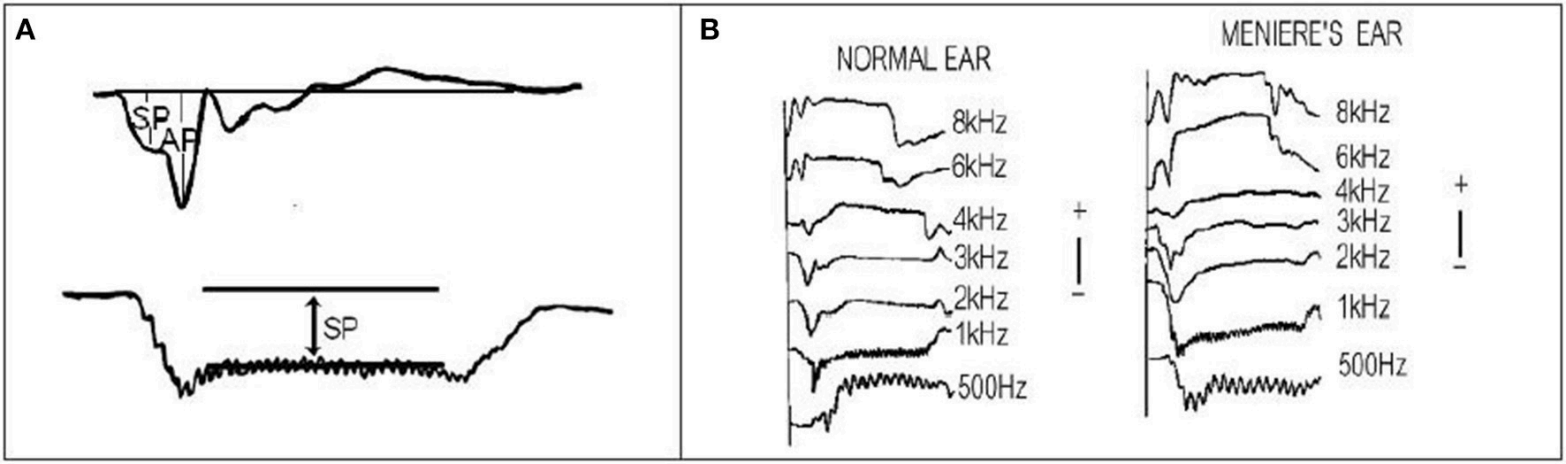
**(A)** Measurement of the click SP/AP ratio. **(B)** Tone burst recordings from a normal ear and an ear affected by Meniere's Disease.

Gibson et al. (1977) were the first to describe an abnormality of the EcochG in ears affected by Meniere's disease. Further studies suggested that the ratio of the SP amplitude and the click action potential (AP) amplitude and was means of identifying Meniere's disease (Figure 5A). Attempts have been made to use extratympanic (ET) electrodes so that non -medical clinicians can undertake the testing but unfortunately the specificity and sensitivity of the SP/AP measurement is poor.

There are several problems using the SP/AP ratio. The size and shape of the AP varies according to the audiogram and the SP also can alter independently. Many of the studies have compared a series of normal hearing ears with ear affected by Meniere's disease with varying hearing losses. The SP/AP ratio only indicates endolymphatic hydrops and not Meniere's disease and it is possible that in the early stages of MD, hydrops may not be present.

The author has published a large series of transtympanic (TT) recordings (Gibson, 2009; Table 1). Meniere's disease was defined using the ASOHNS 1995 criteria (Monsell et al., 1995) and he also used a 10 point scale of 7 or over (Gibson, 1991). The control group had similar hearing loss to the stimulus but on the 10 point scale only scored 1 or less for 0–24 dBHL and less than 2 for greater hearing losses. Endolymphatic hydrops may only be present intermittently in some stages of Meniere's disease, and endolymphatic hydrops has been found in non-Meniere's ears at autopsy so this method of selecting controls has some drawbacks. The results utilizing a click SP/AP ratio stimulus showed poor specificity using this method of selection.

**Table 1:** **The Gibson 10 point score (Gibson, **1991**)**.

Vertigo	Rotational vertigo	1
	Attacks lasting over 10 minutes	1
	Rotational vertigo associated with one or more of: aural fullness, hearing loss/fluctuation, tinnitus	1
Hearing	Sensorineural hearing loss	1
	Fluctuating sensorineural hearing loss	1
	Sensorineural hearing loss associated with one or more of: attacks of vertigo, tinnitus, aural fullness	1
Tinnitus	Tinnitus lasting over 10 minutes	1
	Tinnitus altering with one or more of: hearing fluctuations aural fullness or the attacks of vertigo	1
Aural fullness	Aural fullness lasting over 10 minutes	1
	Aural fullness altering with one or more of attacks of vertigo,tinnitus or hearing fluctuations	1
	Gibson score: one point for each of the above.	Maximum score 10

Using long tone bursts of 8 ms and measuring the amplitude of the SP gave better results (Figure 5B). 1 kHz tone bursts provided the best indication of the presence of endolymphatic hydrops. Table 2 shows the results for 1 kHz. If the results at 500 Hz, 1 kHz, and 2 kHz are combined then the sensitivity of the test reaches 80%, thus 8 out of 10 patients attending the clinic can have the diagnosis of Meniere's confirmed by TT EcochG.

**Table 2:** **Transtympanic EcochG results for 1 kHz tone burst stimuli**.

**1 kHz tonebursts**	**N**	**0–24 dBHL**	**N**	**25–44 dBHL**	**N**	**45–64 dBHL**	**N**	**65–85 dBHL**
Criteria		More negative than +1.7uV		More negative than −6uV		More negative than −4.5uV		More negative than −3uV
True positives	104	98	150	87	182	74	60	47
True negatives	118	108	100	91	55	5	7	6
Sensitivity		94%		58%		41%		78%
Specificity		91.5%		91%		91%		86%
Mann-Whitney *U*-test		0.000		0.000		0.000		0.000

#### Perilymph fistula

**Figure 6:**
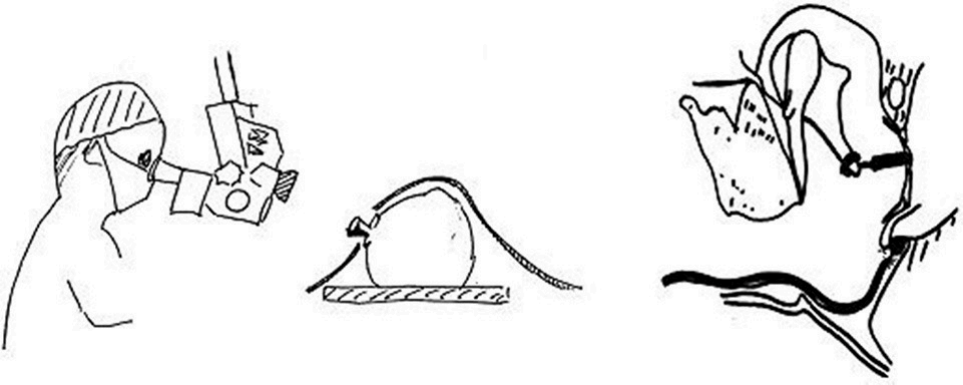
**The arrangement for monitoring middle ear surgery**. The transducer is placed on the operating microscope close to the lens.

Prior to labyrinthectomy, the author made EcochG recordings after perforating the round window membrane during surgery. No obvious change in the waveform was noted until either perilymph was suctioned from the basal coil, or exuded on raising the intrathoracic pressure and then abnormal recordings were obtained until the basal coil refilled. This resulted in a reduced click AP and larger SP giving the waveform a “W” appearance (Figure 6). The original waveform was restored when the perilymph refilled the basal coil.

Based on the intraoperative findings, an attempt was made to devise a test in the clinic. Base recordings were made and then the subject raised the intrathoracic pressure for 20 s and repeated after taking a breath for a further 20 s, the further control recordings were undertaken. Sometimes muscle artifacts contaminated the traces.

The results of this test were disappointing as it no convincing positives were encountered and it seemed that round window perilymph fistulas were either very rare or the test was invalid. Recently some amplitude fluctuations have been encountered in ears affected by a dehiscent superior canal but in these cases no significant change in the SP is noted.

## Introperative electrocochleography

Facial nerve monitoring during delicate ear surgery is mandatory in many countries especially for medico-legal reasons. EcochG can be undertaken during middle ear and cochlear implant surgery and will show subtle changes as well as any inner ear catastrophe.

### Technical aspects

The operating room is full of electrical activity which can interfere with recordings. It is essential that the electrode leads are kept very short and do not act as aerials gathering interference. The author uses a long coax cable which is sterile. The sterile end is given to the surgeon who places an EEG needle electrode in the ear lobe or into the corner of the incision. The reference electrode can be placed anywhere on the patient's body and is coupled with the shielding on the coax cable. The active (non-inverting) electrode is a bendable silver wire which is insulated except for a rounded end. This allows the surgeon to perform the surgery without the electrode getting in the way (Figure 6). The transducer (TDK earphone) is placed near the lens on the operating microscope and the stimulus intensity is calibrated according to the focal length. If possible the high pass filter should remain at 3–10 Hz and the low pass at 3.2 kHz.

### Cochleostomy

**Figure 7:**
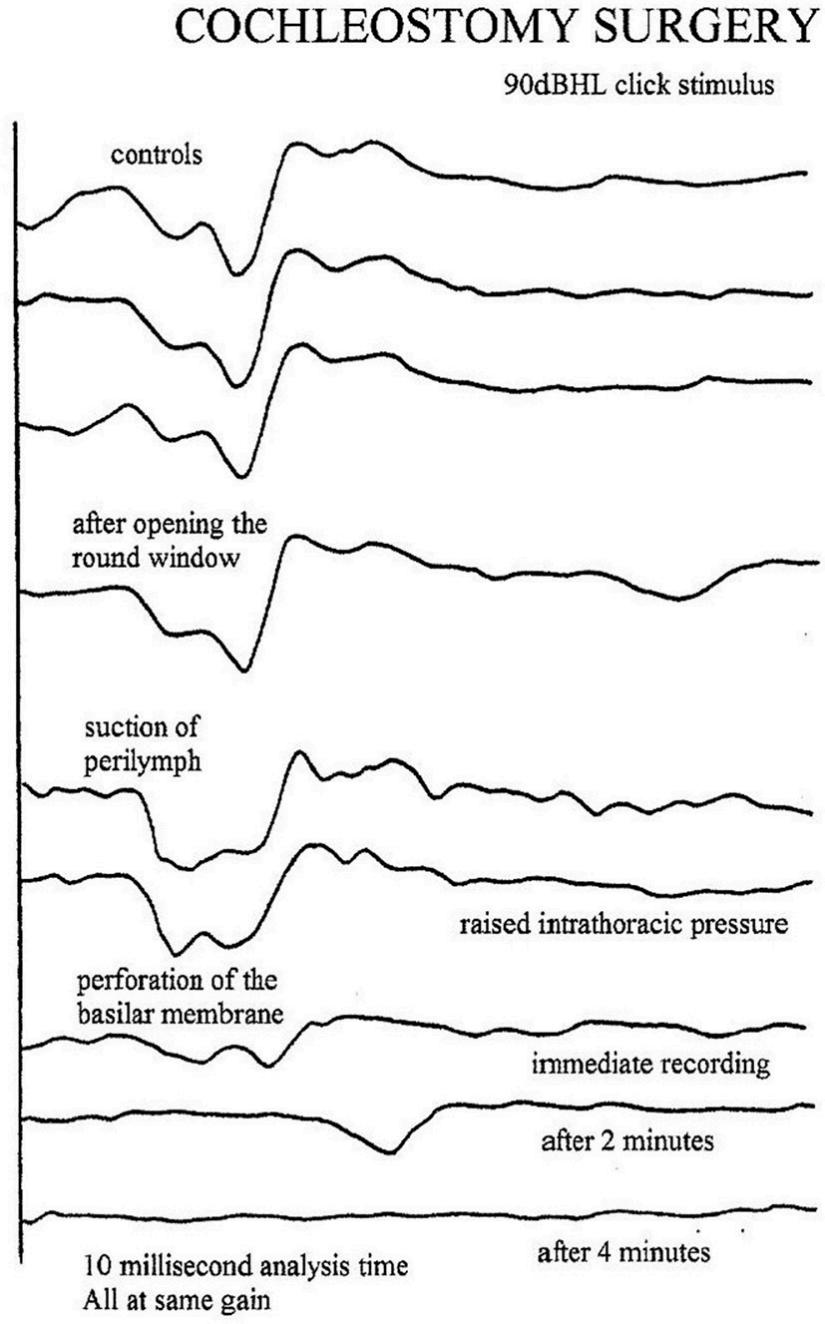
**Click evoked AP after perforation of the basilar membrane (Schuknecht's cochleostomy Procedure; Schuknect, **1982**)**.

This operation for Meniere's disease involved opening the round window and penetrating the basilar membrane with a sharp hook (Schuknect, 1982). This invariably resulted in loss of all residual hearing after 2–3 min. The EcochG changes are shown in Figure 7.

### Stapedectomy

There has always been a dispute as to whether to perform stapedectomy under local anesthesia or general anesthesia. For those who prefer general anesthesia, EcochG monitoring can provide instant feedback similar to the patient's subjective responses (Freeman et al., 2009; Adunka et al., 2016).

### Technique for monitoring stapes surgery

**Figure 8:**
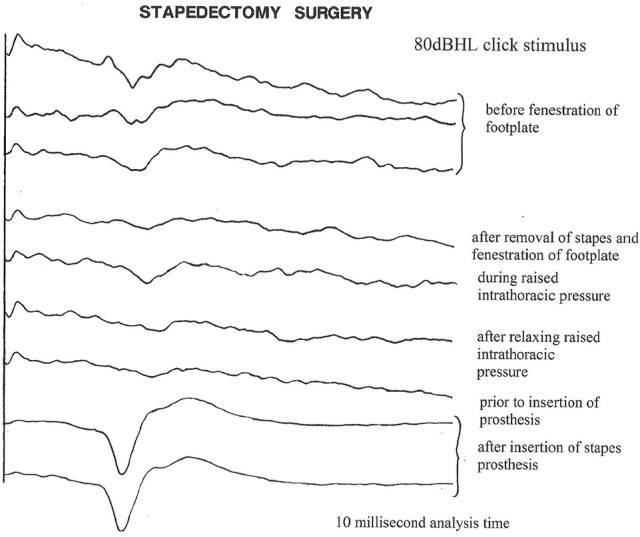
**Click evoked AP during a stapedectomy procedure**.

After tympanotomy, the baseline responses are obtained. Only 20–50 epochs are required at 10–15 per second, so the responses are seen almost instantaneously. After disconnecting the stapes, there is usually a much smaller change than expected, perhaps because of the existing conductive loss. Opening the stapes footplate often shows an improvement. Any suction of the perilymph can show a dramatic change with enlargement of the SP and decrease in the AP (the “W” sign) (Figure 7). The surgeon cam wait and the potentials should recover. After placing the piston, the potentials and the AP threshold improves (Figure 8). Excessive manipulation can cause a deterioration of the AP threshold although the 1 kHz AP is unaffected. In such cases a high frequency audiometric loss can be encountered. post-operatively. The author has one revision case when the EcochG AP was lost on removal of a prolapsed wire fat piston and sadly the hearing was completely lost.

### Perilymph fistula

As mentioned previously, firstly recordings are made before and during raised intrathoracic pressure. If the surgeon sees a possible leak, the site is suctioned and the electrocochleogram observed for the “W” sign. The silver ball can be moved to the oval window when checking the round window for leaks.

### Ossicular chain reconstruction

Immediate benefits of the ossicular chain reconstraction can be monitored but the author prefers to utilize ABR as the silver ball electrode has to be removed on closing the tympanic membrane.

### Cochlear implant surgery

The stimulus transducer (insert earphone) is usually placed in the ear canal. After performing the posterior tympanotomy a silver ball electrode on a flexible wire is placed through the tympanotomy into the round window niche. Recordings can then be acquired to measure any residual hearing. If there is recordable hearing at 500 Hz, 1 kHz, 2 kHz using tone pips or using clicks, the ball electrode is removed from the round window niche and introduced through the atticotomy to lie between the facial nerve and the stapes superstructure. Thus recordings can be made when the implant electrode is inserted through the round window or cochleostomy.

On opening the cochlea through the round window or through a cochleostomy, often an improvement in the CAP threshold of approximately 10 dBHL is often seen. This may be related to an enhancement of the traveling wave. Conversely, if the round window is filled with tissue and gently pressed, a decrease in the CAP threshold is seen.

If the basilar membrane is perforated the CAP is not lost immediately but after 1–2 min. The initial insertion of the electrode usually does not cause any changes even when performed quickly but care has to be taken at 6 mm when the first bend is encountered. Small changes in the CAP suggest a gentler and slower insertion. After full insertion of the electrode, further recordings of the CAP are made to ensure no residual hearing has been lost. Freeman et al. (2009) and Adunka et al. (2016) made recordings before and after insertion of a cochlear implant but found no correlations between the hearing levels recorded immediately after surgery and the audiogram obtained later.

### Labyrinthectomy

The insertion a cochlear implant and labyrinthectomy is becoming a favored means of controlling incapacitating attacks of Meniere's disease. The flexible silver ball electrode is inserted through the posterior tympanotomy and baseline recordings are obtained. The membranous lateral canal is usually removed initially and the abnormal SP disappears and the click SP/AP waveform appears to normalize. It then takes 10–12 min before the CAP disappear.

## Intracochlear recordings

**Figure 9:**
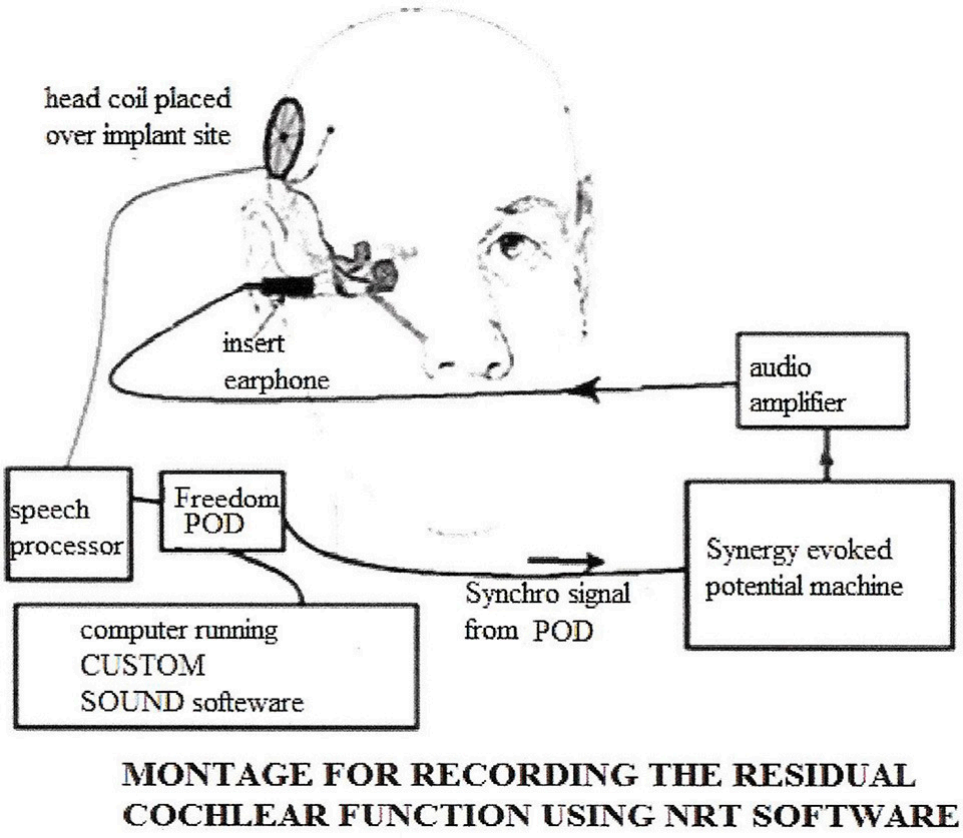
**The montage for recording the intracochlear AP to estimate residual hearing**.

An exciting use of the EcochG has been developed using an electrode on the cochlear implant array. The CM has been used to show sudden changes during the cochlear implant insertion and the CAP can be used to show survival of residual hearing (Figure 9).

### Technical aspects (Figure 9)

Most cochlear implant companies have developed methods of recording electric compound action potentials (ECAP) to measure effect of electrical stimulation. The Cochlear Company has developed neural response telemetry (NRT) and a sophisticated manipulation of the data is required to extrapolate the ECAP from the electrical output of the cochlea implant. The measurement of acoustically evoked potentials is much simpler as there is no electric artifact. The latency of the acoustic response is longer than the electrical and the analysis time has to be extended to 7–10 ms. The acoustic stimulus has to be time locked to the recording apparatus. An insert earphone provides the stimulus.

### Cochlear microphonic recordings

Intracochlear CM recordings in animal studies are very robust and human intracochlear CM recordings can be expected to be equally robust and artifact free. The CAP can take minutes to alter after significant trauma. It is expected that the CM will show sudden changes. so the CM may provide the surgeon with the best indication of intracochlear trauma and hopefully allow the surgeon to alter the insertion to preserve the structures (Campbell et al., 2015).

### Compound action potential recordings

**Figure 10:**
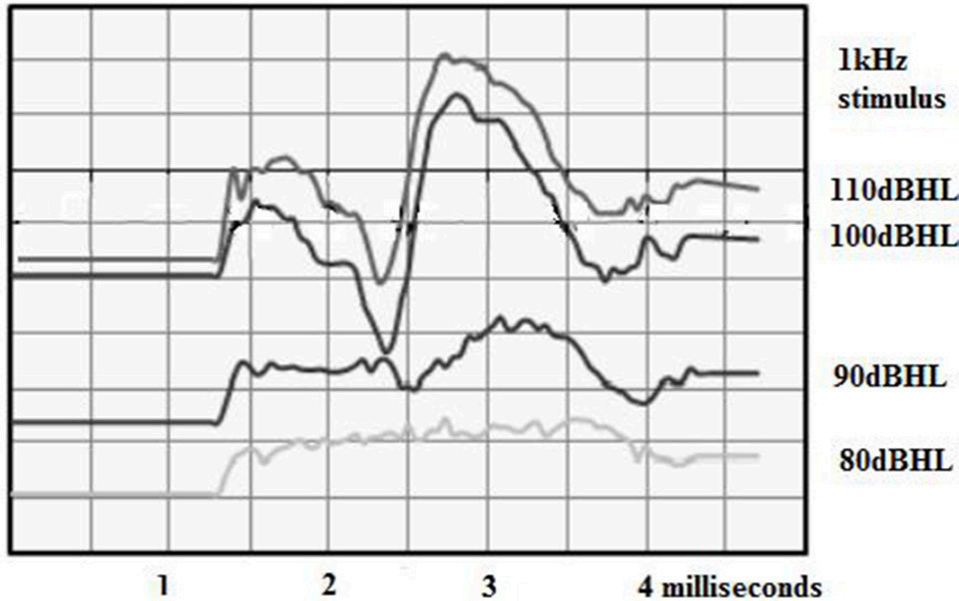
**Intracochlear 1 kHz AP recorded immediately after insertion of a cochlear implant**.

The advantage of CAP is that they give a straightforward indication of the amount of residual hearing as described previously (Figure 10). The advantage of using the intracochlear electrodes and the ECAP platform is that recordings can be obtained at any time after the surgery. A small child could be tested in a free field situation with only the head coil attached. Perhaps these recordings will help to solve the mystery of delayed hearing loss after hearing preservation surgery; for example, the recordings should show if the hearing loss is due to obstruction of the traveling wave or endolymphatic hydrops.

## Conclusions

Although EcochG has been largely ignored, it does have a number of clinical uses ranging from threshold measurements in older difficult to test children, indication of the probability of endolymphatic hydrops as a diagnostic tool for Meniere's disease, and the potential to indicate adverse changes during surgery.

## Author contributions

This article lists the different clinical uses of electrocochleography and provides some detail of the method and apparatus required for each indication. I believe it complements the excellent article by Professor Eggermont in Frontiers 2017.

### Conflict of interest statement

The author declares that the research was conducted in the absence of any commercial or financial relationships that could be construed as a potential conflict of interest. The reviewer WJR and handling Editor declared their shared affiliation, and the handling Editor states that the process nevertheless met the standards of a fair and objective review.
